# Identification of a rice metal tolerance protein OsMTP11 as a manganese transporter

**DOI:** 10.1371/journal.pone.0174987

**Published:** 2017-04-10

**Authors:** Mei Zhang, Baoxiu Liu

**Affiliations:** 1 Provincial Key Laboratory of Applied Botany, South China Botanical Garden, Chinese Academy of Sciences, Guangzhou, China; 2 University of Chinese Academy of Sciences, Beijing, China; National Botanical Research Institute CSIR, INDIA

## Abstract

*M**etal* *t**olerance* *p**roteins* (*MTPs*) are a gene family of cation efflux transporters that occur widely in plants and might serve an essential role in metal homeostasis and tolerance. Our research describes the identification, characterization, and localization of OsMTP11, a member of the *MTP* family from rice. *OsMTP11* was expressed constitutively and universally in different tissues in rice plant. Heterologous expression in yeast showed that *OsMTP11* complemented the hypersensitivity of mutant strains to Mn, and also complemented yeast mutants to other metals, including Co and Ni. Real time RT-PCR analysis demonstrated *OsMTP11* expression was substantially enhanced following 4 h under Cd, Zn, Ni, and Mn treatments, suggesting possible roles of *OsMTP11* involvement in heavy metal stress responses. Promoter analysis by transgenic assays with *GUS* as a reporter gene and mRNA *in situ* hybridization experiments showed that *OsMTP11* was expressed specifically in conducting tissues in rice. DNA methylation assays of genomic DNA in rice treated with Cd, Zn, Ni, and Mn revealed that decreased DNA methylation levels were present in the *OsMTP11* promoter region, which was consistent with *OsMTP11* induced-expression patterns resulting from heavy metal stress. This result suggested that DNA methylation is one of major factors regulating expression of *OsMTP11* through epigenetic mechanisms. OsMTP11 fused to green fluorescent protein (GFP) localized to the entire onion epidermal cell cytoplasm, while vacuolar membrane exhibited increased GFP signals, consistent with an OsMTP11 function in cation sequestration. Our results indicated that *OsMTP11* might play vital roles in Mn and other heavy metal transportation in rice.

## Introduction

The rise of industrialism resulted in the beginning of long-term heavy metal pollution and led to serious environmental problems for modern agriculture and human health. Common heavy metals include Zn, Mn, Ni, Co, Cu, Cd, Pb and Hg, among others. Some of these metals are essential trace elements for numerous physiological processes in plants (Zn, Mn, Ni, Co and Cu), while others are non-essential elements, including Cd, Pb and Hg. However, in large amounts even these essential metals can inactive biomolecules, block functional proteins or displace other essential metal ions and therefore are toxic [1]. In nature, heavy metal stress severely affects plant growth and development, and consequently plants have developed chelation, excretion and subcellular compartmentalization mechanisms to alleviate heavy metal toxicity [2]. In these biological processes, several groups of cation transporters play vital roles in maintaining metal homeostasis and reducing ionic toxicity. In plants, divalent ion transporters include calcium antiporters, cation diffusion facilitator proteins, natural resistance-associated macrophage proteins (NRAMP), and Zn-regulated transporter Fe-regulated transporter like proteins [3]. Among these transporters, cation diffusion facilitator (CDF) family proteins are significant proteins responsible for metal ion homeostasis.

Proteins of the *CDF* family have been cloned from bacteria, fungi, plants, and animals [3], while in plants, these group proteins were renamed as *m**etal* *t**olerance* *p**roteins* (*MTPs*). MTPs are one of the important groups of proteins that function in heavy metal homeostasis in plants and play a critical role in removing metals from the cytosol either by intracellular sequestration into vacuoles/vesicles or by cellular export to excellular region [4, 5]. In general, most of MTPs have the following features in common: an N-terminal signature sequence specific to this family (SLAILTDAAHLLSD); four to six putative transmembrane domains (TMDs); a histidine-rich cytoplasmic loop; a large cation efflux domain; and most of the cytoplasmic C-terminus [6, 7]. Protein sequence alignment and ion-binding specificities classified CDF members from three kingdoms of life (*Archaea*, *Eubacteria*, *Eukaryotes*) into three subgroups: Zn-CDF, Fe/Zn-CDF and Mn-CDF [8].

*ZAT* was the first identified plant cation diffusion facilitator (zinc transporter in *A**rabidopsis* *t**haliana*), which was identified as a crucial gene responsible for Zn tolerance by vacuolar Zn sequestration. *ZAT* was later renamed *AtMTP1* (*M**etal* *T**olerance* *P**rotein* *1* in *A**rabidopsis* *t**haliana*) [9]. *AtMTP1* is expressed constitutively in both roots and shoots and confers a moderate increase in Zn tolerance when over-expressed. Two other metal tolerance protein genes in *Arabidopsis*, *AtMTP3* [10] and *AtMTP11* [11], were also identified. Among these proteins, AtMTP1 and AtMTP3 belong to Zn-CDFs, and AtMTP11 is allied with Mn-CDFs [8]. Function analysis demonstrated that AtMTP11 can specifically transfer Mn [11], and is involved in both Mn tolerance and homeostasis mechanisms in *Arabidopsis*. Another protein in *Stylosanthes hamata*, ShMTP1, is responsible for conferring Mn tolerance in yeast and plants [12]. Recently, several MTPs responsible for Mn transporting have been characterized. The first Mn-CDF member in rice, OsMTP8.1, was identified as tonoplast-localized membrane protein, and its over-expression was found to enhance Mn accumulation and tolerance in yeast and rice [13]. Another rice Mn-CDF member, OsMTP9, was found being involved in the uptake and translocation of manganese in rice plant [14]. Two *Beta vulgaris* Mn-CDF members, BmMTP10 and BmMTP11, and a cucumber CsMTP8, were also found as manganese transporters, and confer Mn tolerance in yeast [15, 16].

Initial analysis of the rice genome identified 10 genes that encode proteins belonging to the *MTP* family [8]. Among these, five of the putative proteins, similar to AtMTP11 and ShMTP1 (belonging to the Mn-CDF group), did not possess the N-terminal signature sequence (SLAILTDAAHLLSD) for the CDF family and lacked a His-rich region that is commonly found in eukaryotic members of the CDF family. Furthermore, the transmembrane prediction program (TMHMM, http://www.cbs.dtu.dk/services/TMHMM/) identified only four membrane-spanning regions in the Mn-CDF subfamily. However, the subfamily does exhibit the typical cation efflux domain in the C-terminus, which mediates metal transport. *OsMTP11* has the highest homology with *AtMTP11* in rice, and its expression is greatly induced by Mn and other heavy metals. Therefore, we propose that *OsMTP11* might be integrally involved in Mn tolerance and other heavy metal homeostasis mechanisms in rice.

Manganese (Mn) is an essential trace element in plants, but it can exert phytotoxic effects at elevated concentrations. In our investigation, a rice cDNA encoding OsMTP11 was characterized at the molecular and biochemical levels. Our results showed that *OsMTP11* complemented the hypersensitivity of a yeast mutant strain to Mn, and complemented partial yeast mutants to other metals, including Co and Ni. Cd, Zn, Ni and Mn treatments induced *OsMTP11* expression. Bisulfite sequence assays showed that a DNA methylation promoter region might regulate the expression pattern of *OsMTP11*. Previously we have identified a new member of rice *MTP* family, *OsMTP1*, which is responsible for the Zn and Cd homeostasis/stress, and mediates the translocation of Zn and Cd from root to the aerial parts in rice [17]. Here in this paper, we characterized *OsMTP11*, and propose *OsMTP11* as a new member of the rice MTP family that serves in Mn and other heavy metal tolerance and homeostasis. In addition, we also characterized the promoter of *OsMTP11* as a heavy metal induced promoter, which could also drive the down-stream genes expressing in conducting tissues in rice.

## Materials and methods

### Phylogenetic and sequence analysis

*MTP* sequences from rice and *Arabidopsis* were identified through a BLAST search of the *Arabidopsis* Information Resource (TAIR; www.arabidopsis.org) and Rice Genome Annotation (http://rice.plantbiology.msu.edu/) or TIGR (www.tigr.org) databases using *AtMTP1* and Cation_efflux (PF01545) domains as queries. Data obtained were predicted amino acid sequences based on nucleotide sequence data. Phylogenetic trees were generated using MEGA 4.0.2. Sequences of *OsMTP11*, *AtMTP11* and *ShMTP1* were aligned using Clustal X 2.0. *OsMTP11* exon prediction was identified in the Rice Genome Annotation Database (http://rice.plantbiology.msu.edu/). The transmembrane prediction of OsMTP11, AtMTP11 and ShMTP1 was performed in the TMHMM program (http://www.cbs.dtu.dk/services/TMHMM/). The *OsMTP11* promoter regions were analyzed in the PlantCARE (http://bioinformatics.psb.ugent.be/webtools/plantcare/html/) program.

### Plant materials, growth conditions and stress treatments

Rice seeds (*Oryza sativa* L. ssp. *japonica* cv. Nipponbare) were sterilized and soaked in water at 28°C for 3 days without light, then transferred to 1/2 MS liquid medium to grow at 28°C in a 16 h / 8 h light cycle for 2 weeks. Different rice organs were harvested at different time periods to isolate total RNAs for *OsMTP11* gene expression analysis. For heavy metal, salt and oxidative stresses, 2-week-old seedlings were transferred into half-strength Murashige Skoog liquid medium plus 0.5 mM CdCl_2_, 5 mM Zn(NO_3_)_2_, 1 mM NiCl_2_, 2 mM MnSO_4_, 300 mM NaCl and 100 μM methyl viologen (MV) respectively for different time periods, and the roots and leaves were subsequently collected to isolate total RNAs or stored at -70°C for further use. Experiments were performed following a completely randomized design with three replications and repeated at least twice. In the rice transgenic assay for *OsMTP11*’s promoter-derived GUS detection, Japonica rice (Zhonghua11) was used according to reference [17].

### Gene expression analysis by real time RT-PCR

Total RNA was extracted with TRIzol Reagent (Invitrogen, USA) and reverse transcribed to cDNA with M-MLV reverse transcriptase (Promega, Shanghai) according to the manufacturer’s instructions. The cDNAs were further used for gene expression analysis. Real time RT-PCR was conducted using a model 7500 real-time PCR system (Applied Biosystems). To normalize the amount of total cDNA present in each reaction, the rice *OsUBQ5* (AK061988) gene was used as an internal control. The primers used for cDNA cloning were as follows: OsMTP11RTF (5’- ACGCTCGTTGTTGTCTGATGGG-3’) and OsMTP11RTR (5’- GTCACTGCAAGCATGATGTCCAC-3’); OsUBQ5RTF (5’- CGCCGTGCTCCAGTTCTA-3’) and OsUBQ5RTR (5’- CGATTTCCTCCTCCTTCCTT-3’), designed according Arvidsson *et al* [18]. In brief, real-time RT-PCR was performed in a total volume of 20 μl reaction mixtures with gene-specific primers. Each reaction included 10 μl of SYBR green real-time PCR master mix (Bio labs), 0.5 μM (each) forward and reverse primers, and 2 μl of cDNA template (equivalent to 100 ng cDNA). The amplification was performed using the following cycle parameters: 94°C for 30 s, followed by 40 cycles of 94°C for 15 s, and 60°C for 40 s for plate reading. Three independent biological repeats were performed for each treatment. Gene expression levels were calculated from the threshold cycle (CT) according to the 2^−ΔΔCT^ method.

### mRNA *in situ* hybridization

mRNA *in situ* hybridization was performed as described by Yuan *et al* [17]. Briefly, the DNA fragment (414 bp) of *OsMTP11* full length cDNA was chosen as a template to generate DIG-labeled probe, which was PCR-amplified with full length cDNA as template, and the primers were as follows: pM11RHF (5’-ATGGGGATGAGTTCAGCTTG-3’) and pM11RHR (5’-AGTAATGGGAGCCAAACGTG-3’). PCR products were then cloned into pGEM-T vector (Promega, Shanghai, China) to generate recombinant plasmid pGOsM11. Transverse sections (8 μm thick) were probed with a digoxigenin-labeled antisense probe or sense probe (control; DIG Northern starter kit; Roche). The slides were observed using a microscope and photographed.

### Promoter cloning and GUS detection

The genomic DNA was isolated from rice leaf (japonica variety Zhonghua 11) with CTAB methods. The promoter region of *OsMTP11* was cloned through standard PCR protocol with genomic DNA as template, and the primers are MTP11PF (5’-CGGAATTCTTATTTGGTTGCCTCCGTGT-3’) and MTP11PR (5’-AACTGCAGGCCATTATTCCACGAACCAG-3’). Then the 1,988 bp promoter fragments were digested with *Eco*RI and *Pst*I, and inserted into the pCAMBIA1381z vector. After it was confirmed by sequencing, the construct was introduced into *Agrobacterium* strain EHA105 and transformed into wild-type Zhonghua 11 following a standard procedure [19]. Histochemical GUS analysis was performed. Briefly, after GUS staining, the rice roots and leaves were decolorized with 70% ethanol, and photographed using a microscope. Or the tissues were fixed with glutaraldehyde solution (0.1M PBS pH7.0, 2% glutaraldehyde, 2.5% PA), embedded with EP812 resin. Then the sections were photographed using a microscope.

### Yeast strains, medium, and transformation

The yeast strains used in this study are summarized in Table 1. The yeast mutants used to derive the parental strain BY4741 were all obtained from Euroscarf (http://www.euroscarf.de/index.php?name=News). Transformants were selected on uracil-deficient medium and grown in synthetic dropout medium containing 2% galactose (SDG medium), including 50 mM (NH_4_)_2_SO_4_, 0.7% yeast nitrogen base without amino acids (YNB, BD Difco, USA), and 0.3% appropriate amino acids, pH6.8. For metal tolerance assays, yeast was grown in liquid SDG medium (with 2% [w/v] galactose) until reaching OD_600_ to 1.0, and drop assays were performed on SDG plates under different concentrations of Mn, Zn, Cd, Co, Ni or Cu. The growth curve of yeast was measured based on the growth rate according to the OD_600_ values.

**Table 1:** List of *S*. *cerevisiae* mutant strains used in this research. Euroscarf (http://web.uni-frankfurt.de/fb15/mikro/euroscarf/) is a collection of single-deletion mutants in *S*. *cerevisiae* (Frankfurt).

Mutant	Background	Mating Type		Genotype	Reference
Wild type	BY4741	MATa	his3Δ1; leu2Δ0; met15Δ0; ura3Δ0		Euroscarf Y00000
*zrc1*Δ	BY4741	MATa	his3Δ1; leu2Δ0; met15Δ0; ura3Δ0; YMR243c::kanMX4		Euroscarf Y00829
*cot1*Δ	BY4741	MATa	his3Δ1; leu2Δ0; met15Δ0; ura3Δ0; YOR316c::kanMX4		Euroscarf Y01613
*ycf1*Δ	BY4741	MATa	his3Δ1; leu2Δ0; met15Δ0; ura3Δ0; YDR135c::kanMX4		Euroscarf Y04069
*pmr1*Δ	BY4741	MATa	his3Δ1; leu2Δ0;met15Δ0; ura3Δ0; YGL167c::kanMX4		Euroscarf Y04534
*smf1*Δ	BY4741	MATa	his3Δ1; leu2Δ0; met15Δ0; ura3Δ0; YOL122c::kanMX4		Euroscarf Y06272
*cup2*Δ	BY4741	MATa	his3Δ1; leu2Δ0; met15Δ0; ura3Δ0; YGL166w::kanMX4		Euroscarf Y04533
*vma8*Δ	BY4741	MATa	his3Δ1; leu2Δ0; met15Δ0;ura3Δ0; YEL051w::kanMX4		Euroscarf Y00292
*vph2*Δ	BY4741	MATa	his3Δ1; leu2Δ0; met15Δ0; ura3Δ0; YKL119c::kanMX4		Euroscarf Y04969

The yeast expression vectors of *OsMTP11* and *AtMTP11* were constructed using two pairs of primers: MTP11YEF (5’- TTTCAGGGCGCCATGGATGGCGGCGGCGGTCGCGGG -3’) and MTP11YER (5’- TCATGCTAGACCATGGCTATTTTTCATGGGACAGAG -3’); and AtMTP11YEF (5’- TTTCAGGGCGCCATGGATGGTTGAGCCAGCTAGTCC -3’) and AtMTP11YER (5’- TCATGCTAGACCATGGCTAACAGTGGGATCTAGCGT -3’) were used to amplify the *OsMTP11* and *AtMTP11* open reading frame regions. The PCR fragments were subsequently subcloned into the *Nco*I sites of pYES260 (Euroscarf), following the GAL1 promoter with the in-fusion technique (BD In-Fusion PCR cloning Kit, Takara), yielding pYES260-OsMTP11 and pYES260-AtMTP11. The pYES260-OsMTP11, pYES260-AtMTP11 and pYES260 were then transformed into yeast cells following Gietz [20].

### Measure of heavy-metal content in yeast

Yeast cells were grown for 36 h in 20 mL of culture (with different metals) and the OD value was measured until OD_600_ to 1.0 was reached. The yeast cultures were then diluted as 1:100 with fresh medium (with total volume of 500 ml medium for one sample). Under liquid-shaking culture for about 24 h (OD_600_ till 1.0), MnSO_4_, CoCl_2_, and NiCl_2_ was supplied separately until the final concentrations of Mn, Co and Ni reached at 3.5 mM, 0.2 mM and 0.15 mM, and yeast cells were allowed to grow for another 24 h to make sure absorbing sufficiently. The cells were collected and washed by centrifugation with double distilled water (ddH_2_O) for three times. Cell pellets were dried in the oven at 65°C for 3–5 days, and then the cell pellets were digested in in 6 mL of nitric acid at 80°C for 1 h following the instructions established by Li and Kaplan [21]. Following digestion, samples were diluted to 100 mL with deionized water and analyzed with Perkin-Elmer Inductively Coupled Plasma Atomic Absorption Spectrometer (ICP-AAS).

### DNA methylation assay by the bisulfite sequence method

Two-week rice seedlings were rinsed separately with 0.5 mM CdCl_2_, 5 mM ZnSO_4,_ 2 mM MnSO_4,_ 1 mM NiCl_2_ and 300 mM NaCl for 72 hrs. The root genomic DNA was subsequently isolated using DNAeasy Plant Mini Kit (Qiagen, Germany). Untreated rice seedlings were used as controls. A total of 1 μg genomic DNA was subjected to bisulfate modification with the EpiTect Bisulfite Kit (Qiagen, Germany) following the manufacturer’s instructions. The *OsMTP11* starting code (ATG) up stream sequence (-2,250 bp) was analyzed on the MethPrimer website (http://www.urogene.org/methprimer/). The primers were designed based on this program, then used to amplify the *OsMTP11* promoter region from -970 to -1216: P1-F: 5’- TGTATTTGAGTTTTATAGTTGTTTTTTT CGG-3’; and P1-R: 5’- AAAATTTTAACTACTTATTTACCATCCTTT -3’. The amplified fragments were subcloned into the pGEM-T Easy vector (Promega) and sequenced. In each sample, 30 clones in total were sequenced and analyzed on Kismeth (http://katahdin.mssm.edu/kismeth/revpage.pl) to identify the DNA methylation status.

### Transient expression of *OsMTP11* in onion cells

DNA fragments containing an open reading frame region without an *OsMTP11* stop codon were obtained via PCR with forward primer MTP11GL: 5’-CGGGATCCATGGCGGCGGCGGTCGCGGG-3’ and reverse primer MTP11GR: 5’-CGGGATCCTTTTTCATGGGACAGAGCGT-3’ (the *Bam*HI site is underlined). The PCR fragment was then integrated into the *Bam*HI site prior to the ATG start code of the Enhanced Green Fluorescent Protein (EGFP) using pUC18-EGFP [22]. The DNA constructs were introduced into onion (*Allium cepa*) epidermal cells as described previously [22]. After incubation at 22°C for 24 h, GFP fluorescence was observed using the confocal laser scanning microscope LSM5 PASAL (Carl Zeiss).

## Results

### OsMTP11 is one of rice *Mn-MTPs*

**Fig 1:**
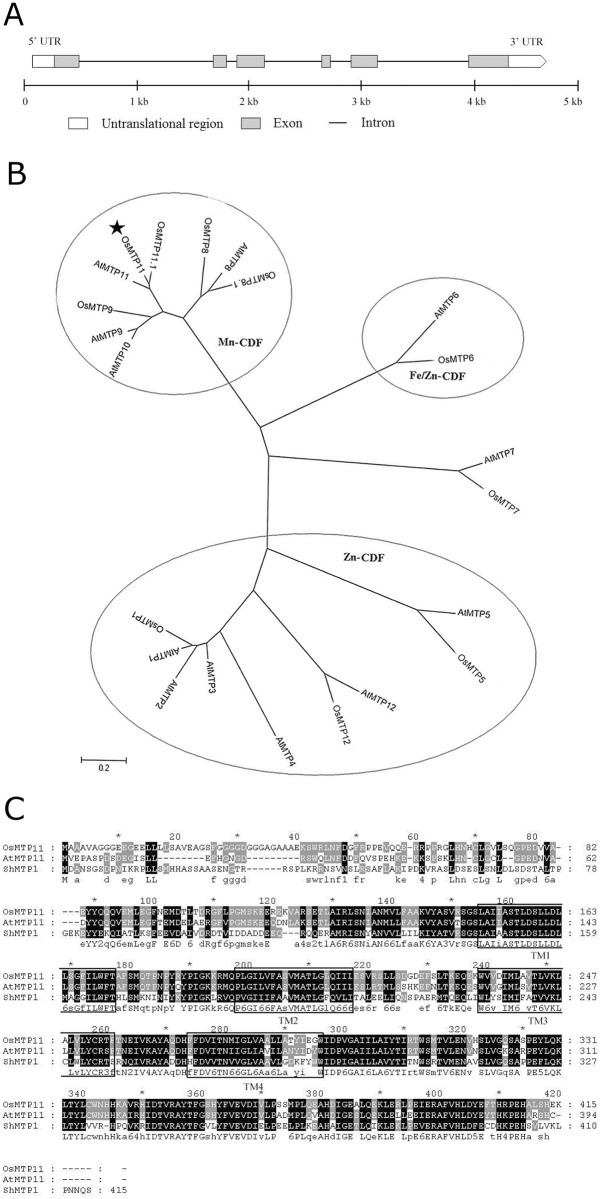
Bioinformatics analyses of *OsMTP11* nucleotide and amino acid sequences. **A.** *OsMTP11* gene structure analysis in the Rice Genome Annotation Database (http://rice.plantbiology.msu.edu/). **B.** Phylogenetic tree of the *MTP* family from rice and *Arabidopsis*. The tree was constructed using MEGA 4.0.2 by the neighbor-joining method. *Arabidopsis* MTP amino acid sequences were obtained from www.tigr.org: AtMTP1, At2g46800; AtMTP2, At3g61940; AtMTP3, At3g58810; AtMTP4, At2g29410; AtMTP5, At3g12100; AtMTP6, At2g47830; AtMTP7, At1g51610; AtMTP8, At3g58060; AtMTP9, At1g79520; AtMTP10, At1g16310; AtMTP11, At2g39450; AtMTP12, At2g04620. Rice MTP amino acid sequences were downloaded from http://rice.plantbiology.msu.edu/. OsMTP11, Os01g62070; OsMTP1, Os05g03780; OsMTP5, Os02g58580; OsMTP6, Os03g22550; OsMTP7, Os04g23180; OsMTP8, Os02g53490; OsMTP8.1, Os03g12580; OsMTP9, Os01g03914; OsMTP11, Os01g62070; OsMTP11.1, Os05g38670; OsMTP12, Os08g32680. **C.** Amino acid alignment of OsMTP11, AtMTP11 [11] and ShMTP1 (AY181256) [12]. Amino acid sequences of four predicted transmembrane (TM) segments are boxed. Amino acid residues with dark shading indicate conserved sequences, and residues with light gray shading indicate those conserved in two protein sequences. **D.** The predicted transmembrane helices of OsMTP11. The transmembrane domains were estimated using TMHMM2: www.cbs.dtu.dk/services/TMHMM/. The peaks show the predicted transmembrane (TM) regions of proteins. These data indicate that OsMTP11 has four obvious TM regions.

The *OsMTP11* gene (Os01g62070) encodes a protein of 415 amino acids (46.1 kDa) containing the cation efflux domain (PF01545, http://pfam.wustl.edu) in its C-terminal region. Members of the cation efflux family are often integral membrane proteins which were found to increase tolerance to divalent metal ions such as Zn, Cd, Mn and Co. Rice genome sequencing results positioned *OsMTP11* on chromosome 1 with six exons and five introns (Fig 1A).

In previous reports [5], *OsMTP11* was grouped into the *Mn-CDF* group. In the present study, phylogeny reconstruction with 10 rice and 12 *Arabidopsis MTPs* showed that *OsMTP11* has the highest homology with *AtMTP11* [23] (Fig 1B). Furthermore, predicted sequence analysis from OsMTP11 protein showed 75.66% and 49.39% identity with AtMTP11 (Loci: At2g39450) and ShMTP1 (AY181256) at the amino acid level, respectively. We also perform *OsMTP11* sequence alignment with *AtMTP11* and *ShMTP1*. The results showed that OsMTP11, like AtMTP11 and ShMTP1, lacks the complete N-terminal signature sequence (SLAILTDAAHLLSD) for the CDF family and exhibits the absence of a His-rich region commonly found in eukaryotic members of the CDF family. Furthermore, the transmembrane prediction program identified models for the OsMTP11 consisting of four (http://www.cbs.dtu.dk/services/TMHMM/) membrane-spanning regions (Fig 1C and 1D). These results indicated that OsMTP11 is a typical Mn-CDF in rice.

### The expression of *OsMTP11* was induced by several heavy metals

**Fig 2:**
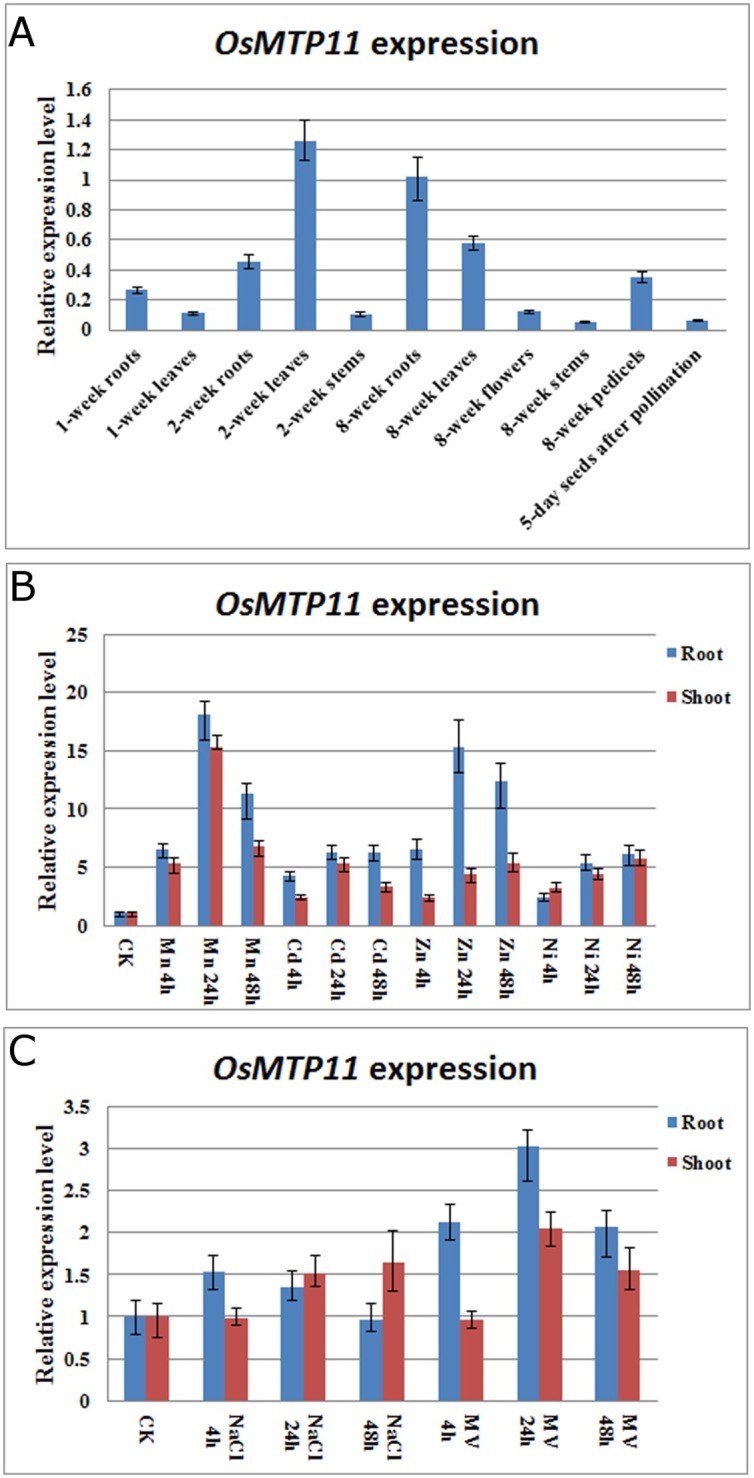
Expression pattern of *OsMTP11* by real time RT-PCR. **A.** Real time RT-PCR results of *OsMTP11* expression in wild-type rice plants (Nipponbare) from different tissues or organs. The amplification of the rice *OsUBQ5* (AK061988) gene was used as a control to normalize the transcript level of *OsMTP11*. **B.** Expression analysis of *OsMTP11* under different heavy metal stresses (Mn, Cd, Zn and Ni) by real time RT-PCR. The expression of *OsMTP11* is increased in rice roots and shoots treated with 0.5 mM CdCl_2_, 5 mM Zn(NO_3_)_2_, 1 mM NiCl_2_, 2 mM MnSO_4_, 300 mM NaCl and 100 μM methyl viologen (MV) for different time periods. **C.** Expression analysis of *OsMTP11* under 300 mM NaCl and 100 μM methylviologen (MV) by real time RT-PCR.

*OsMTP11* spatial expression pattern was investigated by real time RT-PCR analysis. Our results demonstrated that *OsMTP11* was expressed constitutively in most rice tissues (Fig 2A). The highest level of *OsMTP11* transcript was detected in 2-week rice and 8-week roots and leaves. *OsMTP11* was weakly expressed in 1-week rice tissues and 8-week stems, and there were some transcript accumulation in rice florets and immature seeds.

To test if *OsMTP11* expression was affected by heavy metal stresses, transcript accumulation in 2-week rice plants exposed to2 mM MnSO_4_, 0.5 mM CdCl_2_, 5 mM Zn(NO_3_)_2_ and 1 mM NiCl_2_ was assessed by real time RT-PCR analysis. Temporal induction of *OsMTP11* transcripts in response to the above heavy metals was characterized by analyzing roots and leaves at various times following treatment. Fig 2B shows a rapid and marked increase in *OsMTP11* expression following 4 h-stress by Cd, Zn, Ni and Mn, peaking at 24 h after stresses. *OsMTP11* transcript induction was highest due to Mn and Zn treatment compared to Cd and Ni (Fig 2B). Meanwhile, we also checked the expression pattern of *OsMTP11* under salt and MV treatments, our results showed that 300 mM NaCl hardly affected the transcript level of *OsMTP11*, while MV can slightly induce the *OsMTP11*’s expression (Fig 2C).

### Heterogenous expression of *OsMTP11* in yeast confers tolerance to Mn, Co and Ni but not Zn, Cd and Cu

The high sequence homology between OsMTP11 and AtMTP11 at the putative protein level, as well as the observation that *OsMTP11* expression was indeed regulated by several heavy metals suggests that *OsMTP11* might encode a functional cation efflux transporter. Previous reports revealed that AtMTP11 and ShMTP1 specifically transport Mn [11, 12, 23]. Here, OsMTP11 transport abilities for Mn and other divalent ions were also investigated using a series of yeast mutants defective in cation transport.

**Fig 3:**
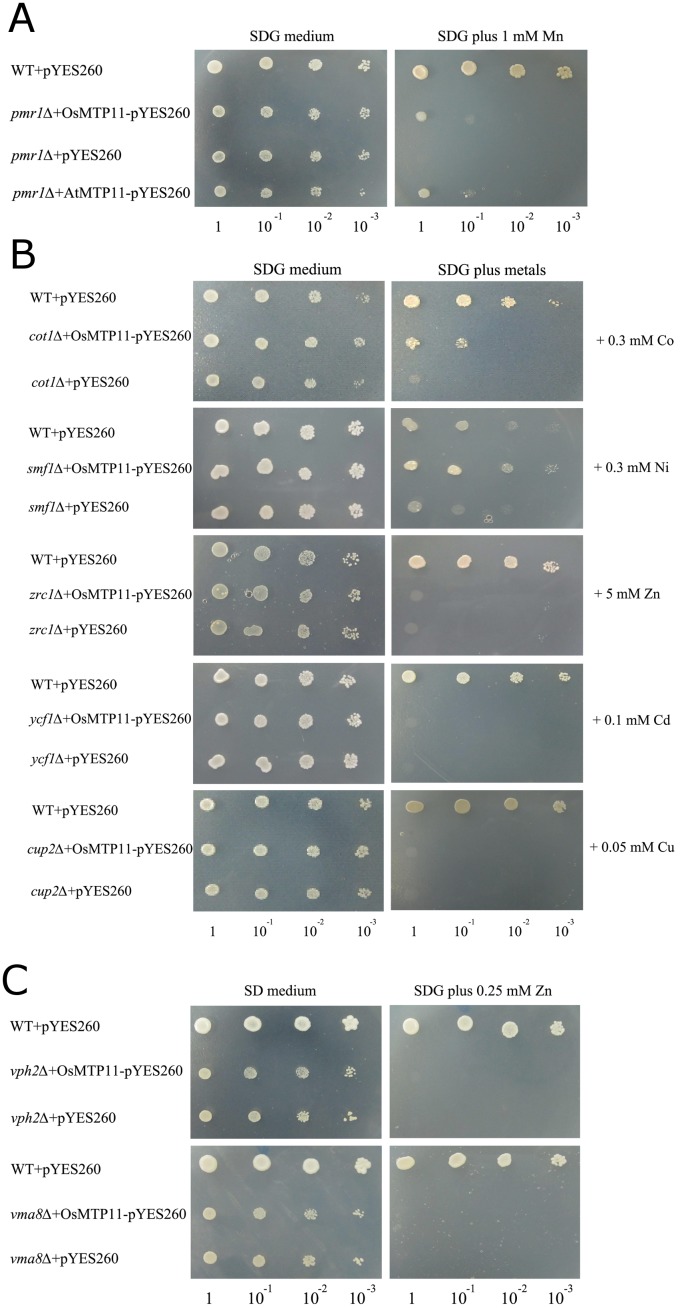
Complementation of yeast mutants on selective medium. **A.** *OsMTP11* and *AtMTP11* genes complement the Mn-hypersensitive phenotype of a *pmr1Δ* yeast mutant on selective medium (SDG medium) without or with 1 mM Mn. **B.** *OsMTP11* complemented the Co-hypersensitive phenotype of the *cot1Δ* yeast mutant and Ni-hypersensitive phenotype of the *smf1Δ* yeast mutant, but could not complement the Zn-hypersensitive phenotype of the *zrc1Δ* yeast mutant, the Cd-hypersensitive phenotype of the *ycf1Δ* yeast mutant and the Cu-hypersensitive phenotype of the *cup2Δ* yeast mutant. **C.** *OsMTP11* is incapable of complementing the yeast vacuolar acidification mutants *vph2Δ* and *vma8Δ*.

First, the Mn-transport ability of the OsMTP11 and AtMTP11 transporters were inferred from growth of *S*. *cerevisiae* Mn-sensitive mutant strain *pmr1*Δ on SDG medium supplemented with 1 mM Mn (Fig 3A). PMR1 is the yeast secretary pathway pump responsible for high-affinity transport of Mn and Ca into the Golgi and confers Mn tolerance by effectively removing Mn from the cytoplasm [24]. Heterologous expression of *OsMTP11*, as well as *AtMTP11*, restored the growth of the yeast mutant strain *pmr1*Δ on 1 mM Mn. The *OsMTP11* and *AtMTP11*-transformed *pmr1*Δ mutant strain exhibited enhanced growth compared to the *pmr1*Δ mutant strain transformed with the empty vector (Fig 3A). Compared with the wild-type yeast, growth of the *pmr1*Δ yeast strain with *OsMTP11* and *AtMTP11* expression was slightly retarded, however the empty vector *pmr1*Δ yeast strain did not grow on 1 mM Mn (Fig 3A).

In addition, we verified whether the *OsMTP11* gene could complement yeast mutants defective in other divalent ion transport (Fig 3B). The *cot1*Δ mutant is hypersensitive to Co [21, 25], and we found that *OsMTP11* partially complemented the *cot1*Δ mutant phenotype when grown on 0.3 mM Co, indicating that OsMTP11 can transport Co to a certain extent. The *smf1*Δ mutant is a Ni sensitive strain. The *SMF1* gene that functions in cellular accumulation of Ni and Mn has been deleted [26]. *OsMTP11* can, to a certain degree, complement the Ni-sensitive phenotype of the *smf1*Δ mutant, suggesting that OsMTP11 also transports Ni. The *zrc1*Δ yeast mutant is a Zn-sensitive strain. In this mutant, the *ZRC1* gene-encoding transporter that sequesters Zn into the vacuole has been deleted [21]. Our results indicated *OsMTP11* could not complement the *zrc1*Δ mutant Zn-sensitive phenotype. We further transformed the *ycf1*Δ, and *cup2*Δ mutant strains, which are unable to grow at the medium plus Cd and Cu, respectively. YCF1 is an ABC transporter that confers Cd tolerance through the transport of Cd conjugates into the vacuole [27]. CUP2 is a yeast copper-binding transcription factor, which can activate transcription of the metallothionein genes in response to elevated copper concentrations [28]. The results showed that *OsMTP11* did not contribute to increased tolerance of *ycf1*Δ to Cd and *cup2*Δ to Cu (Fig 3B).

Two yeast vacuolar acidification mutants (*vma8*Δ and *vph2*Δ) were also chosen to determine if *OsMTP11* could rescue the mutant Zn hypersensitive phenotype. The *VMA8* gene encodes a subunit of the vacuolar-type H^+^-ATPase catalytic domain, and the VPH2 protein is required for functional vacuolar ATPase biogenesis [29, 30]. Yeast mutants *vma8*Δ and *vph2*Δ are unable to acidify their vacuoles and have increased sensitivity to some heavy metals, such as Zn (no growth at 0.25 mM Zn). Our results showed that *OsMTP11* could not restore the growth of *vma8*Δ and *vph2*Δ under 0.25 mM Zn conditions (Fig 3C). This result indicated that OsMTP11 does not assist in the yeast proton gradient in the vacuole. The role of *OsMTP11* is inconsistent with another *MTP*, *PtdMTP1*, which can partially rescue both acidification mutants [31].

**Fig 4:**
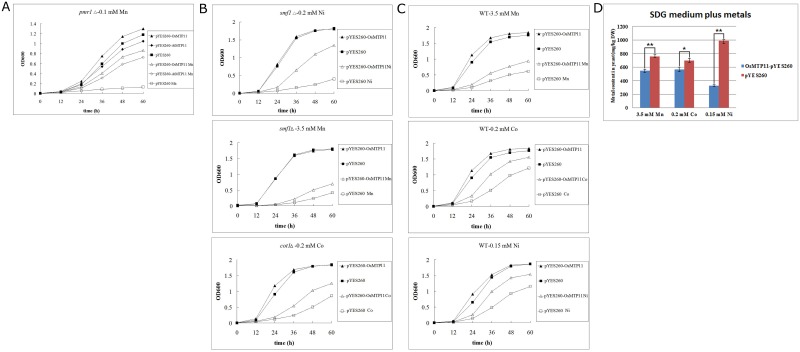
Effect of *OsMTP11* expression in wild yeast BY4741 and yeast mutants for heavy metal tolerance and cation content. **A.** Yeast mutant (*pmr1Δ*) transformed with the empty plasmid vector (pYES260) or *MTP* genes in pYES260 (*OsMTP11* and *AtMTP11*) were grown in either SDG liquid medium or SDG liquid medium supplemented with 0.1 mM Mn^2+^. Culture optical density at 600 nm (OD_600_) was determined at 12 h intervals. **B.** Yeast mutants (*smf1Δ* and *cot1Δ*) transformed with the empty plasmid vector (pYES260) or the *OsMTP11* gene in pYES260 were grown in either SDG liquid medium or SDG liquid medium supplemented with 0.2 mM Ni^2+^ (*smf1Δ*), 3.5 mM Mn^2+^ (*smf1Δ*) and 0.2 mM Co^2+^ (*cot1Δ*). Culture optical density at 600 nm (OD_600_) was determined at 12 h intervals. **C.** Wild type yeast BY4741 transformed with the empty plasmid vector (pYES260) or the *OsMTP11* gene in pYES260 was grown in either SDG liquid medium or SDG liquid medium supplemented with 3.5 mM Mn^2+^, 0.2 mM Co^2+^ and 0.15 mM Ni^2+^. Culture optical density at 600 nm (OD_600_) was determined at 12 h intervals. **D.** *OsMTP11* expression affects heavy metal content in yeast. Wild type yeast BY4741 transformed with the empty plasmid vector (pYES260) or the *OsMTP11* gene in pYES260 was grown in SDG liquid medium supplemented with 3.5 mM Mn^2+^, 0.2 mM Co^2+^ and 0.15 mM Ni^2+^. The yeast was harvested at an OD_600_ value as 1.5~1.8, then the heavy metal was measured with ICP-AAS. Results represent means ± standard error of three biological replicates. Single asterisk symbols (*) indicate significant differences between control (empty vector pYES260) and experimental samples (OsMTP11- pYES260) at an individual detection (0.01 < P < 0.05); Double asterisk symbols (**) indicate significant differences between control (empty vector pYES260) and experimental samples (OsMTP11- pYES260) at an individual detection (P < 0.01).

The yeast growth curve was recorded under different heavy metal treatments. First, we evaluated mutant strain *pmr1*Δ under 0.1 mM Mn stress. Results showed that cell growth of *pmr1*Δ transformed with an empty vector was inhibited completely, whereas growth of *pmr1*Δ cells expressing *OsMTP11* was nearly restored at 0.1 mM Mn, as same as *AtMTP11* (Fig 4A). These data indicated that OsMTP11 complemented the Mn sensitivity of the *pmr1*Δ mutant yeast, acting as a Mn transporter on similar manners with AtMTP11. We also chose yeast mutant *smf1*Δ to investigate growth conditions under 3.5 mM Mn and 0.2 mM Ni stress. Our results showed that *OsMTP11* partially restored the restrained growth condition of *smf1*Δ by Mn and Ni (Fig 4B). Similarly, *OsMTP11* partially restored the restraining conditions of *cot1*Δ by 0.2 mM Co (Fig 4B), which indicated that OsMTP11 could transport Co and elevate the tolerance of Co in *cot1*Δ yeast.

A previous study revealed that AtMTP1 actively transported Zn^2+^ into the vacuole using a pH gradient [32], and AtMTP3 conferred Zn tolerance in yeast cells through intracellular Zn sequestration [10]. We determined the basis of *OsMTP11*-mediated heavy metal tolerance by analyzing Mn, Co and Ni accumulation in the yeast transformants. First, we measured the growth curve of wild yeast strain BY4741 heterologous expression of *OsMTP11* under Mn, Co and Ni treatments. Our results showed that all of the above metals inhibited yeast cell growth, and *OsMTP11* partially restored yeast growth rates (Fig 4C). Subsequently, we analyzed Mn, Co and Ni accumulation in yeast (Fig 4D). Results showed that yeast transformed with pYES260-OsMTP11 accumulated lower levels of Mn, Co and Ni than yeast cells transformed with the empty vector pYES260. This suggests that *OsMTP11*-mediated Mn, Co and Ni tolerant yeast cells are derived from extracellular excretion of heavy metal cations.

### DNA methylation of the *OsMTP11* promoter region may regulate *OsMTP11* induced-expression under heavy metal stresses

**Fig 5:**
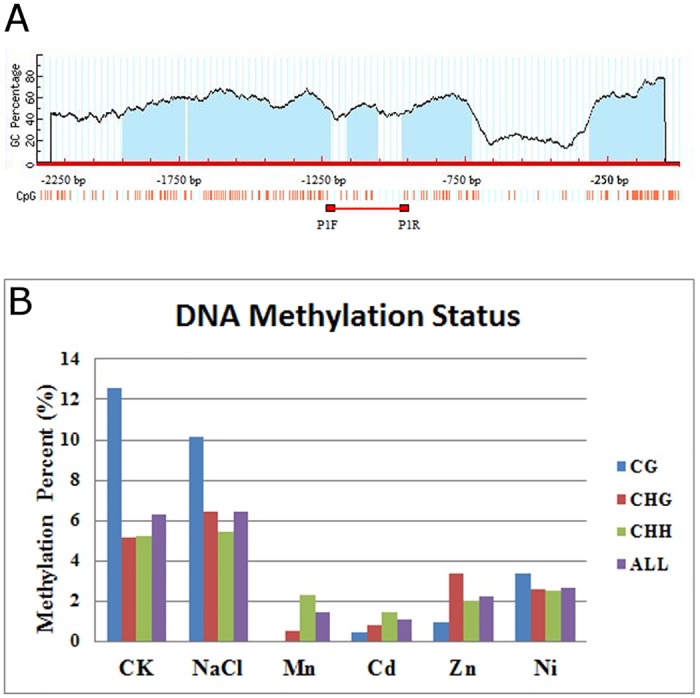
DNA methylation analysis of the *OsMTP11* promoter region. **A.** Schematic distribution of CpG sites and CpG islands in the *OsMTP11* promoter region. Blue area indicates CpG islands. The scale bar indicates 100 bp. Red bar indicates the region analyzed by bisulfate sequencing. **B.** Comparison of percentages of cytosine methylation of three different types (CpG, CpHpG, and CpHpH) in rice seedling roots between control and under heavy metal stress (0.5 mM CdCl_2_, 5 mM ZnSO_4,_ 2 mM MnSO_4,_ 1 mM NiCl_2_) or salt treatment (300 mM NaCl) for 72 hours.

DNA methylation is an epigenetic regulatory mechanism of gene expression. Abiotic stresses, such as aluminum, heavy metals, and water are known to alter cytosine methylation throughout the genome and at specific loci [33]. In this study, *OsMTP11* expression was induced by Cd, Zn, Ni and Mn and *OsMTP11* promoter sequence (-2,250 bp) analysis indicated that five CpG islands exist in the promoter region (Fig 5A), which can mediate DNA methylation at cytosine and further affect gene expression. We investigated the epigenetic regulatory mechanism of *OsMTP11* induced by heavy metals through performing DNA methylation detection assays using sodium bisulfate treatments and DNA sequencing. Our results showed a decreased methylation rate at CG, CHG, and CHH sites in the *OsMTP11* promoter region under Cd, Zn, Ni and Mn treatments (Fig 5B), suggesting induced *OsMTP11* expression by heavy metal stress might be regulated by promoter DNA methylation. While compared to salt treatment (300 mM NaCl), under which the expression level of *OsMTP11* could not be affected (Fig 2B), accordingly, the DNA methylation status kept similar with the control (Fig 5B).

### OsMTP11 functions as a cation transporter and is localized in the cytoplasm

**Fig 6:**
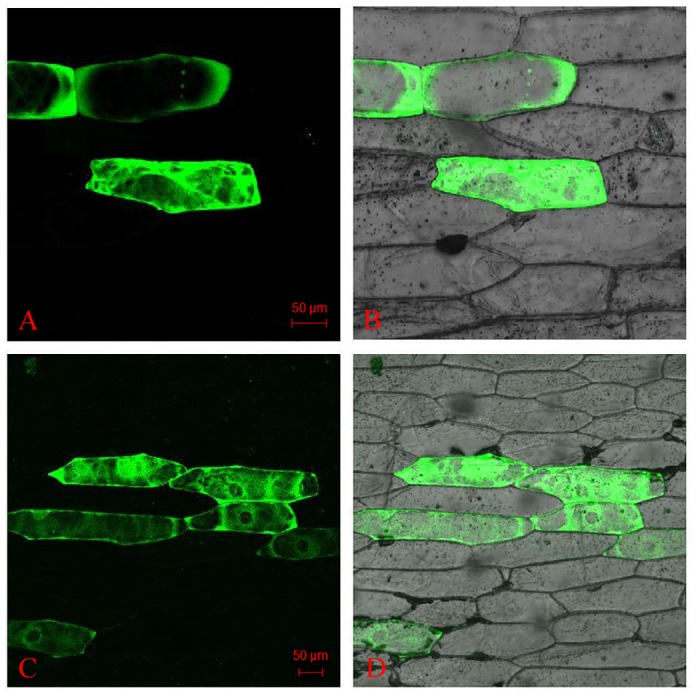
Transient expression of OsMTP11::EGFP in onion epidermal cells. **A.** Localization of EGFP control. **B.** Bright-field and fluorescence merged image of A. **C.** Localization of OsMTP11::EGFP. **D.** Bright-field and fluorescence merged image of C.

The subcellular localization of OsMTP11 was determined by an *in vivo* targeting experiment in which OsMTP11-fused soluble enhanced fluorescent protein (EGFP) was transiently expressed in onion (*Allium cepa L*.) epidermal cells. The results localized OsMTP11-EGFP primarily to the entire cytoplasm (Fig 6, lower panels). However it was clearly absent in the vacuole, but the vacuolar membrane exhibited strong GFP signals. This was obviously distinguishable from the EGFP control, which was detected throughout the cell (Fig 6, upper panels). Based on these *in vivo* targeting results, we concluded that rice OsMTP11 is predominantly localized to the cytoplasm endomembrane system.

The OsMTP11 subcellular localization results were verified by bioinformatics analysis with four different programs to predict the possible localization sites of OsMTP11 (Table 2). Predictions from WoLF PSORT (http://wolfpsort.org/) showed that OsMTP11 localization at the plasma membrane, endoplasmic reticulum, endoplasmic reticulum vacuolar membrane and vacuolar membrane scored respectively 5, 4.5, 4.5 and 3.5, which indicated that OsMTP11 may evenly distribute at the plasma membrane and endomembrane systems, and coincided with our EGFP fusion protein assay results. Similarly, the Plant-Ploc (http://www.csbio.sjtu.edu.cn/bioinf/plant/) program predicted OsMTP11 localized in the cytoplasm; and Target P (http://www.cbs.dtu.dk/services/TargetP/) predicted a low OsMTP11 score value for a chloroplast transit peptide (0.211, cTP), mitochondrial targeting peptide (0.053, mTP) and secretory pathway signal peptide (0.157, SP), with uncertain localization scores (0.715). ARAMEMNON (http://aramemnon.botanik.uni-koeln.de/) predicted OsMTP11 distributed primarily in secretory pathways with a high absolute value (3.7). Cumulatively, the above program predictions indicated OsMTP11 with cytoplasmic localizations, primarily in the plasma membrane and endomembrane system, serving a role in transporting divalent ions extracellularly or into intracellular vacuoles.

**Table 2:** Subcellular localization of OsMTP11 was predicted using different databases and programs.

MTP	WoLF PSORT^A^	Plant-PLoc^B^	Target P^C^	ARAMEMNON^D^
**OsMTP11**	**plasma membrane (5.0)**	**Cytoplasm**	**cTP (0.211)**	**chloroplast (0.0)**
	**endoplasmic reticulum (4.5)**		**mTP (0.053)**	**mitochondrion (0.0)**
	**endoplasmic reticulum vacuolar membrane (4.5)**		**SP (0.157)**	**secr.pathways (3.7)**
	**vacuolar membrane (3.5)**		**Other (0.715)**	

The numbers in parenthesis indicate the prior probability that such protein localizes to a given site.

^A^WoLF PSORT (http://wolfpsort.org/) predicts the subcellular localization sites of proteins based on both known sorting signal motifs and some correlative sequence features such as amino acid content.

^B^Plant-Ploc (http://www.csbio.sjtu.edu.cn/bioinf/plant/) is a program for predicting plant protein subcellular location. The predictor includes 11 subcellular locations in cell: (1) Cell wall, (2) Chloroplast, (3) Cytoplasm, (4) Endoplasmic reticulum, (5) Extracellular, (6) Mitochondrion, (7) Nucleus, (8) Peroxisome, (9) Plasma membrane, (10) Plastid and (11) Vacuole.

^C^Target P (http://www.cbs.dtu.dk/services/TargetP/) predicts the subcellular location of proteins based on the predicted presence of any of the N-terminal presequences: chloroplast transit peptide (cTP), mitochondrial targeting peptide (mTP) or secretory pathway signal peptide (SP).

^D^ARAMEMNON (http://aramemnon.botanik.uni-koeln.de/) is a database about transmembrane (TM) proteins and transporters in *Arabidopsis*, rice, cyanobacteria, and organellar genomes; it enables direct comparison of the predictions of seven different TM span computation programs and the predictions of subcellular localization by eight signal peptide recognition programs.

### *OsMTP11* was expressed mainly in conducting tissues in rice

**Fig 7:**
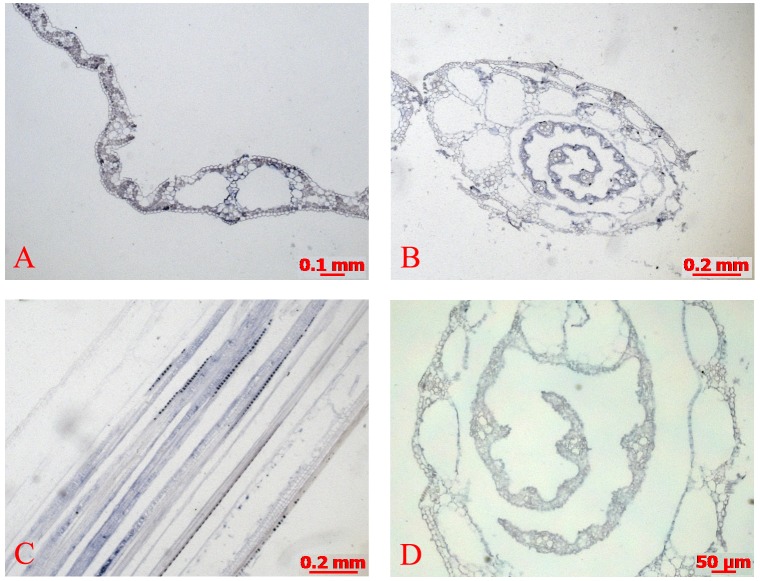
mRNA *in situ* hybridization of *OsMTP11* in rice leaves. *Blue* or *purple* precipitates indicate positive *OsMTP11* mRNA signals. **A.** Transverse sections of a mature leaf. **B.** Transverse sections of a young leaf bud. **C.** Longitudinal sections of a seedling leaf bud. **D.** Control with the transverse sections of leaf bud.

Due to OsMTP11 as a transporter protein, we also detected the expression pattern of *OsMTP11* through mRNA *in situ* hybridization and promoter derived- GUS staining assays. Our results indicated that *OsMTP11* transcripts distributed mostly in conducting tissues in rice, and has a similar pattern with *OsMTP1* [17]. In Fig 7, *OsMTP11* mRNA accumulated specifically near vascular tissue (Fig 7A, 7B and 7C). The cross sections of leaf and sheath showed that the blue signals were distributed in specific sieve tube files (A and B), while the longitudinal leaf sheath sections showed that the signals were continuous (C). No positive reaction above background levels was detected on sections hybridized with sense RNA (Fig 7D).

**Fig 8:**
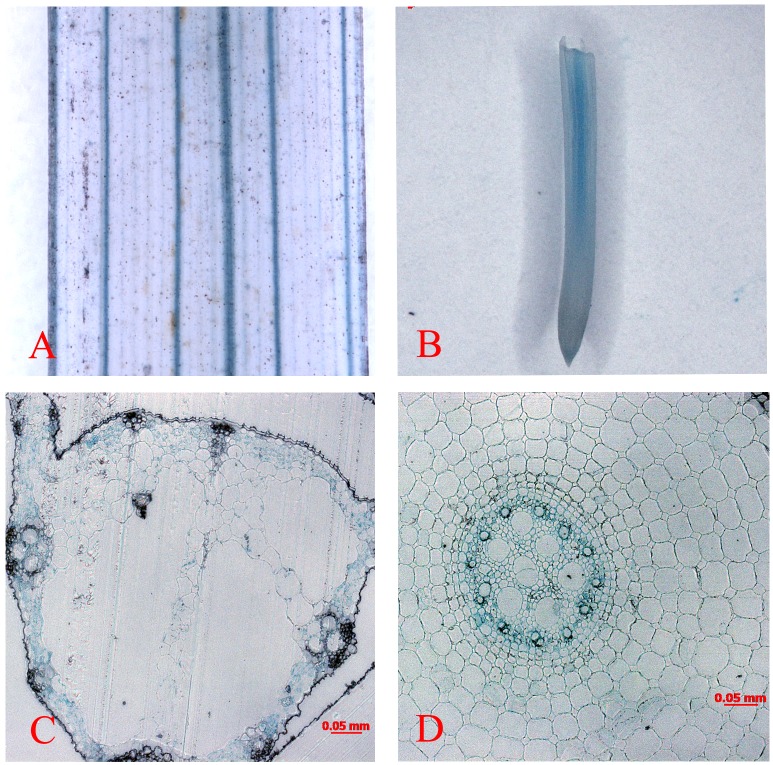
*OsMTP11* native promoter-drived GUS staining in rice leaves and roots. Blue precipitates indicate positive GUS signals. **A.** Leaf with GUS staining in vein tissue. **B.** Root tip with GUS staining in conducting tissue. **C.**Transverse sections of A. **D.** Transverse sections of B.

To further explore the expression pattern of *OsMTP11*, the putative promoter, about 2 kb DNA upstream of the *OsMTP11* coding region sequence, was fused to the GUS reporter gene. Histochemical staining for GUS activity in transgenic rice plants showed that *OsMTP11* was constitutively expressed, and the higher-intensity GUS staining was observed in conducting tissues in the rice leaves and roots (Fig 8).

### The *OsMTP11* promoter contains regulatory elements in response to environmental signals

**Fig 9:**
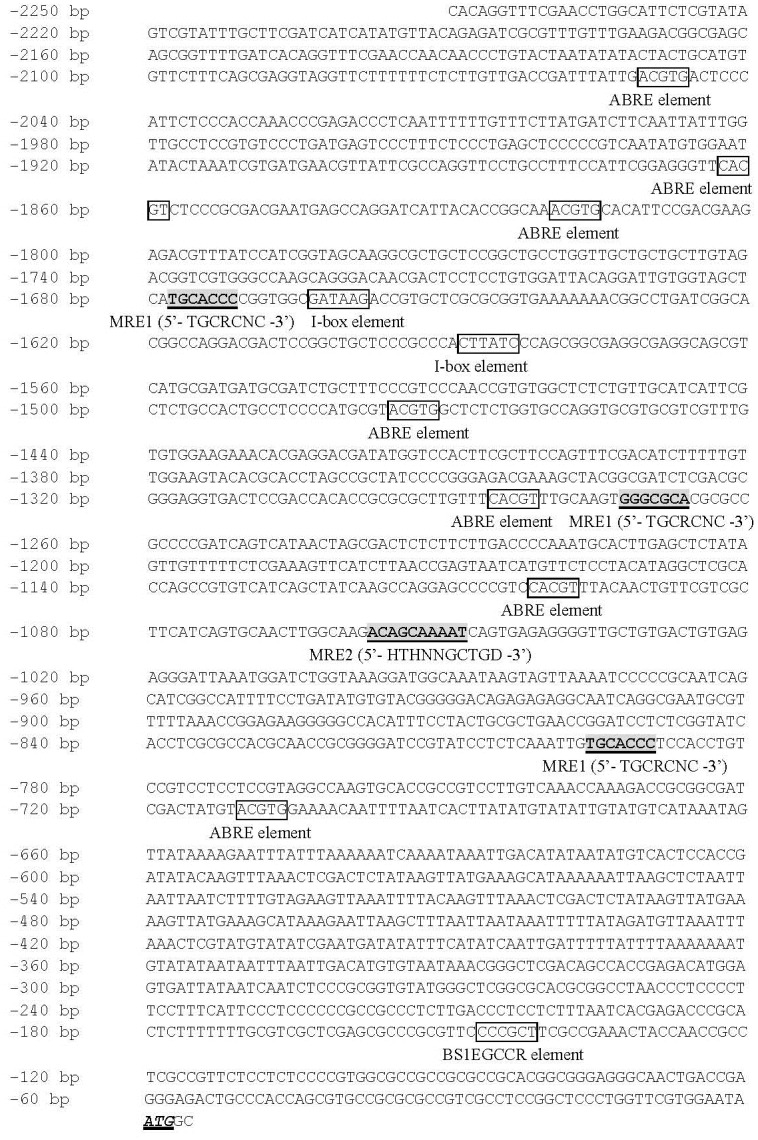
The *OsMTP11* putative promoter region (-2,250 bp) sequence. The transcription start site is denoted +1, and the putative start codon is underlined. Diagram of the *OsMTP11* promoter region using PlantCARE (http://bioinformatics.psb.ugent.be/webtools/plantcare/html/) showed the presence of a number of potential cis-acting elements that respond to environmental signals. MRE, metal-response element [34, 35]; ABRE, abscisic acid-response element [36]; I-box, light-response element [37]; BS1EGCCR, "BS1 (binding site 1)" found in CCR gene promoter, which is a cis-element required for vascular expression of the cinnamoyl CoA reductase gene in *E*. *gunnii* [38]. MREs include MRE1: 5’-TGCRCNC-3’ (R = A or G; N = any residue) [34] and MRE2: 5’-HTHNNGCTGD-3’ (D = A, G, or T; H = A, C, or T; N = any residue) [35].

In this study, Northern blot analysis characterized *OsMTP11* as a heavy metal-induced gene. Therefore, analyzed the *OsMTP11* promotor region (a 2,250 bp upstream genomic DNA region corresponding to the ATG start codon) to identify cis-acting regulatory elements, which can be regulated by heavy metal and other environmental stresses. The *OsMTP11* promoter region (-2,250 bp to ATG) is shown in Fig 9. Analysis of the *OsMTP11* promoter using PlantCARE (http://bioinformatics.psb.ugent.be/webtools/plantcare/html/) indicated that a number of potential cis-acting elements that respond to environmental signals were present (Fig 9), and these elements are classified in Table 3. Two types of heavy metal-responsive elements (MREs) have been identified and their corresponding metal-induced transcription factors have been characterized in detail. One kind of MRE consists of a highly conserved heptanucleotide core (MRE1; 5’- TGCRCNC -3’, R = A, G; N = any residue) and less conserved flanking nucleotides [34]; the other is comprised of the consensus sequence 5’- HTHNNGCTGD -3’ (MRE2; D = A, G, or T; H = A, C, or T; N = any residue) [35]. We found 3-MRE1s and 1-MRE2 in the *OsMTP11* promoter region. They were localized separately at -796 bp, -1,273 bp, -1,678 bp and -1,058 bp sites, which may be involved in heavy metal induced *OsMTP11* expression. Seven putative abscisic acid-response element-like motifs (ABRE; 5’- ACGTG -3’) were present at -711 bp, -1,102 bp, -1,286 bp, -1,476 bp, -1,820 bp, -1,863 bp and -2,051 bp. These elements could be responsible for gene expression during dehydration and other stress [36]. Two I-box elements (5’- GATAAG -3’) thought to be involved in light response were also found [37] at -1,589 bp and -1,664 bp. Among others, there was also a vascular-specific expression cis-element (BS1EGCCR, 5’- AGCGGG -3’) [38] at -147 bp site, indicated that *OsMTP11* was expressed in rice vascular tissue, and played a crutial role in ion transporting. These putative regulatory elements suggest that the promoter region of *OsMTP11* may respond to a variety of environmental signals, including heavy metal and other forms of stresses.

**Table 3:** The statistics for MREs (Metal Response Elements), ABREs (Abscisic acid-Response Elements), I-box elements (light response elements) and BS1EGCCR (cis-element required for vascular-specific expression, Binding Site 1 found in CCR gene) in *OsMTP11* promoter (-2,250 bp upstream to translational start coden).

Cis-acting elements	Number	Locations
MRE1 (5’-TGCRCNC-3’)	3	-796; -1,273; -1,678
MRE2 (5’-HTHNNGCTGD-3’)	1	-1,058
ABRE (5’-ACGTG-3’)	7	-711; -1,102; -1,286; -1,476; -1,820; -1,863; -2,051
I-box (5’-GATAAG-3’)	2	-1,589; -1,664
BS1EGCCR (5’-AGCGGG-3’)	1	-147

## Discussion

The aim of this study was to clarify the biochemical mechanisms of heavy metal transport, particularly Mn, by OsMTP11 and possibly elucidate the biological roles of *OsMTP11*, especially from the point of expression pattern affected by heavy metal. Rice is the primary grain crop in China and a large part of its planting area is polluted by heavy metals, including Cd, Ni, Mn, Cu, and Zn, among others. In recent years [4, 39], the mechanisms of heavy metal’s absorption, translocation and accumulation by plants has attracted much research interest and been studied comprehensively, especially in crop plants. Metal transporters are key factors which mediate the first step of plants responding to heavy metals, and then affect the physiological and biochemical mechanism of metal detoxification in plant. Cation diffusion facilitator (CDF), also renamed as metal tolerance protein (MTP) in plants, is a sort of key metal transporters and play pivotal roles in metal absorption and translocation [40]. Here in this paper, we described a rice metal tolerance protein gene, *OsMTP11*, using the following approaches: bioinformatics, expression pattern, yeast biochemical mechanisms, and protein subcellular localization analysis. These studies provided much needed data on the functions of OsMTP11 in heavy metal (especially Mn) sequestration in rice plants.

### OsMTP11 is a Mn transporter but not specific

Sequence analysis revealed that *OsMTP11* displayed many of the characteristic features of *Mn-CDF* ion transporters, such as *AtMTP11* [23] and *ShMTP1* [12], suggesting a similar molecular mechanism and physiological function among these genes. In this study, heterologous expression of *OsMTP11* in *S*. *cerevisiae* cells provided a viable experimental system to evaluate *OsMTP11* heavy metal transport function. Our results showed that *OsMTP11*, similar to *AtMTP11*, complemented Mn-sensitivity in the yeast mutant *pmr1Δ*, indicating that OsMTP11 had the capability of Mn-transport in yeast. In addition, *OsMTP11* partially complemented Co- and Ni- sensitivity of the yeast mutants *cot1Δ* and *smf1Δ*, suggesting OsMTP11 could also transport these cations. However, its metal-specific transport feature in plant cells remains unclear and requires further investigation. When *OsMTP11* was expressed heterologously in wild type yeast BY4741, it also increased yeast tolerance to Mn, Co and Ni. Accordingly, Mn, Co and Ni content was substantially reduced in yeast, which indicated that *OsMTP11* might mediate cation excretion and consequently elevate heavy metal tolerance in yeast.

The cation diffusion facilitator (CDF) family is a ubiquitous family of heavy metal transporters and widely distribute in all three kingdoms of life (Archaea, Eubacteria, Eukaryotes) [8]. Based on sequence alignment and bioinformatics analysis, and coupled with the substrate specificities of some characterized CDF members, Montanini *et al* [8] classified the family members (273 proteins in total) into three major groups (Zn-, Fe/Zn- and Mn-CDF), and some members’ substrate specificity have been tested in yeast. Then Kolaj-Robin *et al* [40] further classified the CDF family (including 329 proteins in total) into 18 groups and uncovered novel clades (III and XII) with undefined substrate specificity (Ni^2+^ and Co^2+^). Studies have shown that the substrate specificities of CDF members usually depend on some conserved amino acid residues in transmembrane region [8, 23, 40]. Due to heavy metal cations often belong to transition metal elements and possess similar atomic structure, some CDF members might recognize and bind similar metal cations, and result in the non-specificity of substrate recognition.

### The promoter of *OsMTP11* is tissue-specific and metal-inducible

Real time RT-PCR analysis was used to examine the primary expression patterns of *OsMTP11* and assess the possible roles of *OsMTP11* during plant growth and development. Our results revealed that *OsMTP11* was predominantly expressed in mature leaves, roots and stems. These results demonstrated that *OsMTP11* mRNA was mainly distributed in actively transporting and vigorously metabolizing tissues during rice growth and development, and the expression pattern coincided with a possible function of cation transportation in plants. OsMTP11 was identified as a Mn/Ni transporter, and we didn’t find it could transport Zn and Cd in functional complementation assay by yeast mutants, while at the expression level in rice, the transcription of *OsMTP11* was still induced by different metals including Mn, Cd, Zn and Ni. As to Mn, Zn and Ni, which are all essential trace elements for plant growth, while Cd as non-essential, toxic element, we performed the metal treatments with different concentration, such as relatively higher concentrations for Mn (5 mM), Zn (2 mM) and Ni (1 mM), and a lower concentration for Cd (0.5 mM). The expression of *OsMTP11* was strongly up-regulated by heavy metal Cd, Zn, Ni and Mn stress, which indicated that *OsMTP11* was involved in metal tolerance and homeostasis at the expression level in rice. We also analyzed the *OsMTP11* promoter region sequence, with potential cis-acting elements to respond to heavy metal and environmental signals. Promoter derived GUS staining assay and mRNA *in situ* hybridization results showed that *OsMTP11* was mainly expressed in conducting tissues in rice, and its expression pattern had obvious tissue-specific feature. Our results indicated that the native promoter of *OsMTP11* was heavy metal inducible and vascular bundle specific. The expression pattern of *OsMTP11* was coinciding with the biological role that OsMTP11 transport the metal ion intracellularly or intercellularly.

The promoter features of genes were greatly involved in the gene’s biological functions since the expression patterns were decided by the promoter region, and the recognition of plant promoters often occurs in specific tissues or under physiological stress conditions [41]. The results in this paper suggested that the promoter of *OsMTP11* belongs to a metal induced promoter, linking heavy metal specific cellular responses in rice. Our results were consistent with previous reports regarding *BjMTP1* [42] and *BjCET2* [43]. Ni, Cd or Zn was shown to activate *BjMTP1* expression and *BjCET2* was strongly up-regulated by Cd and Zn stress, but not by low temperature, salt and drought.

### The DNA methylation status of promoter region affected the metal-induced expression pattern of *OsMTP11*

Furthermore, we detected some DNA methylation sites (CpG islands) in *OsMTP11* promoter regions. Our results showed that heavy metal stress (such as Cd, Zn, Ni and Mn) can reduce the DNA methylation rate in the promoter region, and DNA methylation in the *OsMTP11* promoter may be one crucial epigenetic mechanism that regulates *OsMTP11* gene expression.

DNA methylation is an important epigenetic factor and exists extensively in plant genomes. It has two major roles: defending the genome against invasive DNA and gene expression regulation [44]. Heavy metal stresses can affect gene expression in plants, and the promoter region is a key factor that influences expression patterns [45]. The epigenetic regulation of gene expression through cytosine methylation in the promoter region under stresses will supply significant evidence that plants respond to abiotic stress. In plants, DNA methylation patterns are sensitive to abiotic stress, and can contribute to heritable stress adaptation that correlates with changes in genome methylation [46]. There are three specific cytosine methylation types: CpG, CpHpG, and CpHpH (H = A, T, or G), which are often catalyzed by different DNA methyltransferases [47]. In *Arabidopsis*, stress-induced transgenerational responses can alter DNA methylation, and cause higher tolerance to stress in untreated progeny [46]. When *Taraxacum* plants were exposed to different ecological stresses, considerable methylation variation was observed throughout the genome, which resulted in increased epigenetic variation among offspring [48]. DNA methylation of promoter region is an epigenetic modification that plays an important role in gene regulation in response to abiotic stress, and growing evidences from recent studies have suggested that demethylation of promoter region could enhance the expression of genes when plants exposed to stresses [49]. *TaGAPC1* is a multiple stress responding gene, and its’ expression were induced by salt and dehydration, along with the demethylation of promoter region of *TaGAPC1* by the bisulfite sequencing PCR (BSP) detection [50] These studies revealed that in plants, DNA methylation is a type of gene expression regulation manner, and plays a pivotal role in response to abiotic stress.

## Conclusions

In summary, our results indicate that OsMTP11 is a Mn-transport protein that drives the metal efflux to the yeast extracellular compartment, and might also be an important Mn transporter in rice. The expression pattern analysis indicated that *OsMTP11* was induced by heavy metals (such as Mn, Cd, Zn, Ni), and the promoter region of *OsMTP11* contains several metal response elements (MREs), which might play vital roles in regulating the metal inducible expression pattern. Combining that DNA methylation status detection of CpG island in promoter region, this research further proved the epigenetic regulation of *OsMTP11* expression was metal-specific. A detailed biological function study of *OsMTP11* should be conducted in future research by plant transgenic experiments. The results of the present study offer insights into a Mn molecular pathway and other heavy metal absorption, transport and reposition processes in rice.
